# SeqVerify: An accessible analysis tool for cell line genomic integrity, contamination, and gene editing outcomes

**DOI:** 10.1016/j.stemcr.2024.08.004

**Published:** 2024-09-12

**Authors:** Merrick Pierson Smela, Valerio Pepe, Steven Lubbe, Evangelos Kiskinis, George M. Church

**Affiliations:** 1 Wyss Institute at Harvard University, Boston MA, USA; 2 The Ken & Ruth Davee Department of Neurology and Department of Neuroscience, Feinberg School of Medicine, Northwestern University, Chicago, IL, USA; 3 Simpson Querrey Center of Neurogenetics, Feinberg School of Medicine, Northwestern University, Chicago, IL, USA; 4 Department of Genetics, Harvard Medical School, Harvard University, Cambridge, MA, USA

**Keywords:** stem cell, pluripotent stem cell, whole-genome sequencing, microbial contamination, aneuploidy, genome editing, single-nucleotide polymorphisms, software, quality control, SeqVerify

## Abstract

Over the last decade, advances in genome editing and pluripotent stem cell (PSC) culture have let researchers generate edited PSC lines to study a wide variety of biological questions. However, abnormalities in cell lines such as aneuploidy, mutations, on-target and off-target editing errors, and microbial contamination can arise during PSC culture or due to undesired editing outcomes. The ongoing decline of next-generation sequencing prices has made whole-genome sequencing (WGS) a promising option for detecting these abnormalities. However, this approach has been held back by a lack of easily usable data analysis software. Here, we present SeqVerify, a computational pipeline designed to take raw WGS data and a list of intended genome edits, and verify that the edits are present and that there are no abnormalities. We anticipate that SeqVerify will be a useful tool for researchers generating edited PSCs, and more broadly, for cell line quality control in general.

## Highlights

• SeqVerify is a tool for analyzing whole-genome sequencing data from cell lines
• SeqVerify validates gene edits and detects aneuploidy, mutations, and contamination
• SeqVerify is easily installed from Bioconda and runs on both Linux and MacOS

## Teaser

Pierson Smela, Pepe, and colleagues developed SeqVerify, a computational tool for analyzing whole-genome sequencing data from genome-edited cell lines. Starting from raw sequencing reads and a list of intended edits, SeqVerify validates on-target edits and detects abnormalities such as aneuploidy, mutations, and contamination. SeqVerify is easily installable from Bioconda and will be a useful tool for cell line quality control.

## Introduction

Pluripotent stem cells (PSCs) have found important uses in many areas of biological research, and their ability to differentiate into a variety of cell types has enabled the development of cell-based therapies derived from PSCs. Gene editing technologies such as CRISPR-Cas9 have enabled the engineering of PSC lines containing specific alleles of interest, such as disease-relevant mutations or fluorescent reporters.

However, over the years, researchers have identified several common abnormalities that can arise during PSC culture (Andrews et al., 2022; Ludwig et al., 2023). First, PSCs can become aneuploid due to chromosomal rearrangements or mis-segregation. The most frequent aneuploidies in PSC cultures involve chromosomal or sub-chromosomal duplications (Assou et al., 2020; Taapken et al., 2011), and some of these, such as gain of 20q11.21, have been characterized to affect the phenotypes of the cells (Markouli et al., 2019; Nguyen et al., 2014). Such aneuploidies are relatively common, affecting roughly 12% of tested PSC lines on average (Taapken et al., 2011), with the frequency increasing over long-term passaging.

Second, PSCs can gain point mutations, which can be enriched during prolonged culture due to providing a growth advantage. For example, the tumor suppressor genes *TP53* and *BCOR* are recurrently mutated in PSCs (Merkle et al., 2017; Rouhani et al., 2022). Although mutation rates in PSCs are not abnormally high (Merkle et al., 2022; Thompson et al., 2020), harmful mutations can often be present in somatic cells used to derive induced PSCs (Rouhani et al., 2022), or can occasionally arise during PSC culture (Merkle et al., 2017). In addition to posing potential safety risks for PSC-derived cell therapies, genetic variation in PSCs can influence the outcomes of cell differentiation *in vitro* (Arthur et al., 2024), affecting the reproducibility of experiments.

Third, as with other cell cultures, PSC cultures can be contaminated with microbes such as *Mycoplasma*. This is a relatively common problem; a study in 2015 was able to detect sequencing reads mapping to *Mycoplasma* in 11% of mammalian cell culture datasets in the NCBI Sequence Read Archive (Olarerin-George and Hogenesch, 2015). Good cell culture practice, including recurrent testing, is essential for avoiding contamination.

Furthermore, gene editing of PSCs can introduce additional abnormalities. At the on-target site, undesired editing outcomes may be present. Traditional PCR-based genotyping can sometimes fail to detect these outcomes when they involve a large insertion of plasmid or mitochondrial DNA into the target site (Simkin et al., 2022). Although off-target editing is usually rare (Veres et al., 2014), the rate may be greatly increased when less specific editing tools, such as APOBEC-based cytosine base editors, are used (McGrath et al., 2019; Zuo et al., 2019).

In order to ensure valid experimental results and the safety of PSC-derived therapeutics, it is important to detect these abnormalities and choose PSC lines without them. Existing quality control methods, including karyotyping, SNP arrays, and quantitative PCR, typically focus on detecting one particular type of abnormality (Assou et al., 2020). However, the ongoing decline of next-generation sequencing prices has made whole-genome sequencing (WGS) an effective quality control option. Notably, WGS is an all-in-one detection method for any abnormality involving changes to DNA sequences such as aneuploidy or mutations, presence of unwanted sequences such as plasmid integration or *Mycoplasma*, or cell line misidentification. WGS data can also help select PSC lines for experiments based on polygenic risk scores for traits of interest (Merkle et al., 2022).

Yet until now, WGS analysis has required considerable expertise in bioinformatics due to a lack of easily usable software. Although other researchers have used WGS for PSC quality control (Merkle et al., 2022), this only looked at wild-type cell lines and did not analyze the effects of genome editing, and the analysis pipeline code was not publicly available. Here, we present a computational pipeline, SeqVerify, that analyzes short-read WGS data for quality control of wild-type or edited PSCs. SeqVerify can validate on-target genome editing, find the insertion sites of untargeted transgene integrations, and detect mutations, aneuploidies, microbial contamination, and misidentification. SeqVerify provides an end-to-end analysis framework, with simple inputs (raw WGS data and a list of intended edits) and easily interpretable outputs. We have made our pipeline easily installable via Bioconda. Furthermore, we showcase the performance of SeqVerify on a set of knockin human induced PSC (hiPSC) lines generated in our lab and benchmark SeqVerify relative to previous results on a set of independently edited hiPSC lines (Simkin et al., 2022). We anticipate that WGS and SeqVerify will be a valuable quality control method for researchers working with PSCs, and more broadly, for cell line quality control in general.

## Results

### The SeqVerify pipeline

#### Overview of the Seqverify pipeline

**Figure 1:**
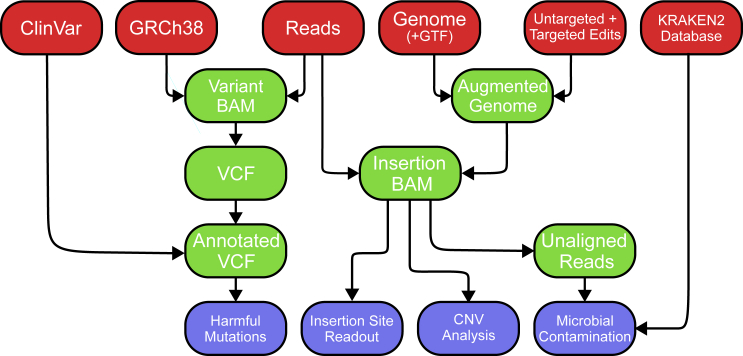
A flowchart showing the steps involved in the SeqVerify pipeline In red, the possible types of inputs that the pipeline can take for the types of analysis that can be performed. In light green, the major intermediate files produced during the running of the pipeline, output in the non-temporary output folder. In light blue, the four major outputs of the pipeline: the insertion site readout and CNV analysis graphs and binaries, as well as the microbial contamination and harmful mutation readouts if the KRAKEN and SNV analysis portions of the pipeline are enabled, respectively.

SeqVerify is an end-to-end pipeline that performs a variety of quality control functions (Figure 1). First, SeqVerify generates an “augmented genome” from a reference genome and a user-provided list of targeted edits and/or untargeted transgene insertions. SeqVerify will then align the raw WGS reads to this augmented genome, validate edits, detect insertion sites, and analyze copy-number variation (CNV). SeqVerify also detects microbial contamination using KRAKEN2 (Wood et al., 2019). Additionally, SeqVerify will align the reads to a wild-type reference genome, detect and filter single-nucleotide variants (SNVs), and annotate them using the ClinVar database (Landrum et al., 2018). Finally, if two or more samples are analyzed using SeqVerify, the SNVs can be automatically compared. This is useful for detecting cell line misidentification or for identifying SNVs arising during cell culture or editing that were not present in the original cells.

#### Installation

SeqVerify was developed for and tested on Linux systems including Windows Subsystem for Linux. It can also run on MacOS or other Unix-based systems. SeqVerify can be installed using the conda package manager, and it can be downloaded from Bioconda with the following command: conda install -c bioconda seqverify.

We recommend this installation method since it also comes pre-packaged with all the dependencies needed to run. However, it can also be downloaded from GitHub with dependencies installed separately.

In terms of technical specifications, any system powerful enough to run BWA-MEM in reasonable time will also be acceptable for SeqVerify, and there are options available for multithreading and limiting memory usage that permit users to tune SeqVerify to their needs. The overall runtime using 20 threads on an Intel Xeon Processor E5-2683 v4 (40M Cache, 2.10 GHz) is approximately 11–12 h per sample at 10X genome-wide coverage, increasing proportionally with increasing sequencing depth. SeqVerify was slightly faster (7–8 h) using 16 threads on an M3 Max MacBook Pro laptop. Detailed usage instructions for SeqVerify are provided in File S1.

#### Automatic download of reference data

The seqverify --download_defaults command will automatically download all the default files for a standard analysis of human cells. These are:

(1) T2T-CHM13v2.0 as the overall reference genome (Nurk et al., 2022; Rhie et al., 2023),
(2) GRCh38 (primary assembly) as the reference genome for SNV calling,
(3) PLUSPF 8 GB as the default KRAKEN2 database,
(4) ClinVar as the default VCF annotation database,
(5) snpEff.config as a fresh snpEff configuration file should the user want to manually specify advanced snpEff options.

SeqVerify downloads all of them from their respective FTP servers and stores them in a seqverify_defaults folder in the working directory where the command is run. If, when running the pipeline, certain options are left blank (reference genome, KRAKEN database, etc.), SeqVerify will automatically attempt to use these from the seqverify defaults folder to correctly run the pipeline.

#### Validation of edits at known target sites

**Figure 2:**
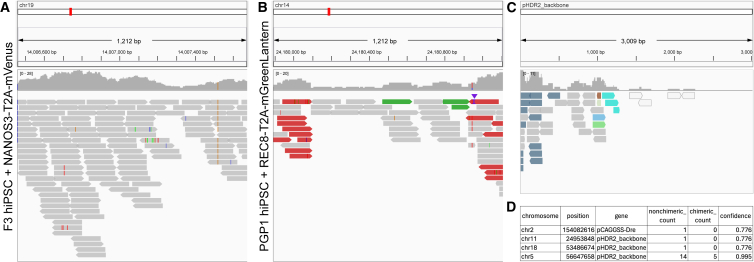
Detection of targeted and untargeted insertions using SeqVerify hiPSCs with fluorescent reporter knockins (A: NANOS3-T2A-mVenus; B: REC8-T2A-mGreenLantern; C and D: DDX4-T2A-tdTomato) were analyzed by WGS and the SeqVerify pipeline. IGV plots were automatically generated showing the insertion and 200 bp of flanking wild-type sequence on either side. (A) The NANOS3 reporter line is a homozygous knockin. A SNP present in the starting cells is visible (brown line). (B) By contrast, the REC8 reporter line is heterozygous; read pairs highlighted in red by IGV denote a “deletion” of the T2A-mGreenLantern sequence on one of the alleles. (C) The automatically generated IGV plot shows reads aligning to the homology-directed repair (HDR) donor plasmid backbone. Reads highlighted in blue have their mates mapped to the human genome. (D) Automatic detection of the plasmid insertion site (at the *DDX4* locus on chr5).

To validate edits at known sites, SeqVerify takes an input file listing genomic coordinates and DNA sequences to be inserted, edited, or deleted at those coordinates. SeqVerify will use this information to generate an edited reference genome, corresponding to the user’s intended edits. After aligning reads to this genome using BWA-MEM (Li and Durbin, 2009), SeqVerify will automatically generate figures using Integrative Genomics Viewer (IGV) (Thorvaldsdóttir et al., 2013), displaying the genomic coordinates provided by the user and showing the aligned reads. Since SeqVerify saves the BAM file output after alignment, the user can also manually open this file in IGV if more detailed inspection is desired. We tested SeqVerify on hiPSC lines that we edited with fluorescent protein reporter knockins at loci such as *NANOS3* and *REC8*. On-target homozygous edits (Figure 2A) are easily distinguishable from undesired outcomes (Figure 2B). In total, we have performed WGS and SeqVerify analysis on 14 knockin hiPSC lines generated in our lab (Table 1). Notably, we detected three instances of undesired plasmid integrations into the editing site that were missed by PCR-based genotyping.

**Table 1:** Editing outcomes in 14 knockin hiPSC lines that were previously genotyped by PCR and Sanger sequencing

Parental line	Edits	On-target results	Plasmid integrations
F3 hiPSC	FOXL2-tdTomato; NR5A1-mGreenLantern	*FOXL2* homozygous, *NR5A1* heterozygous (indel on non-edited allele)	None
F2 hiPSC	DDX4-tdTomato-PuroTK	*DDX4* homozygous	Off-target partial insertion of Cas9/gRNA plasmid
F2 hiPSC	DDX4-tdTomato, DAZL-mGreenLantern (clone #1)	*DDX4* homozygous, *DAZL* homozygous	On-target insertion of HDR plasmid backbone in one allele of *DAZL*
F2 hiPSC	DDX4-tdTomato, DAZL-mGreenLantern (clone #2)	*DDX4* homozygous, *DAZL* homozygous	None
F66 hiPSC	DDX4-tdTomato (clone #1)	*DDX4* homozygous	None
F66 hiPSC	DDX4-tdTomato (clone #2)	*DDX4* homozygous	None
PGP1 hiPSC	DDX4-tdTomato	*DDX4* homozygous	On-target insertion of HDR plasmid backbone in one allele of *DDX4*
F3 DDX4-tdTomato hiPSC	NANOS3-mVenus	*DDX4* homozygous, *NANOS3* homozygous	None
F3 DDX4-tdTomato hiPSC	REC8-mGreenLantern	*DDX4* homozygous, *REC8* homozygous	None
F3 DDX4-tdTomato hiPSC	TFAP2C-mGreenLantern	*DDX4* homozygous, *TFAP2C* homozygous	None
F3 DDX4-tdTomato hiPSC	NPM2-mGreenLantern; PiggyBac transposon insertions for LHX8, SOHLH1, ZNF281, and FIGLA overexpression	*DDX4* homozygous, *NPM2* homozygous	Ten distinct transposon integrations detected
F3 DDX4-tdTomato hiPSC	SYCP3-mGreenLantern	*DDX4* homozygous, *SYCP3* homozygous	On-target insertion of HDR plasmid backbone at *SYCP3*
PGP1 DDX4-tdTomato hiPSC	REC8-mGreenLantern	*DDX4* homozygous, *REC8* heterozygous	None (except the *DDX4* insertion from the parental line)
PGP1 DDX4-tdTomato hiPSC	SYCP3-mGreenLantern	*DDX4* homozygous, *SYCP3* homozygous	None (except the *DDX4* insertion from the parental line)

#### Detection of untargeted transgene insertions

SeqVerify will also accept untargeted transgene sequences as input. This is useful for finding the insertions of transposons and lentiviruses, or for detecting inadvertent integration of plasmids used in editing or induced PSC (iPSC) reprogramming. User-provided sequences are appended to the reference genome and treated as extra chromosomes during alignment. Similarly to targeted insertions, SeqVerify will display reads aligning to these transgenes using IGV. We tested SeqVerify for the ability to detect undesired integration of our gene editing plasmid backbone in edited cells, an abnormality which is common yet easily missed by standard PCR genotyping (Simkin et al., 2022). In one of our cell lines edited at the *DDX4* locus, we observed plasmid integration into the target site (Figure 2C).

However, manually looking through alignments to detect insertion sites is tedious and does not scale well. Therefore, SeqVerify will also automatically find insertion sites by detecting junctions between transgenes and the host genome. After reads have been aligned to the modified reference genome, SeqVerify will parse the output SAM file, extract chimeric read pairs aligning to a transgene and to a host chromosome, and output a comma-separated text file listing any detected insertion sites (Figure 2D, File S3). In addition to providing the number of detections per site, SeqVerify also calculates a confidence score from 0 to 1, based on a Poisson likelihood calculation (Lander and Waterman, 1988), which reflects how likely it is that the insertion is actually present and not an artifact of parts of the genome that look similar to the selected transgene sequences. For details of how this score is calculated, see the supplementary information. SeqVerify’s insertion site detection also automatically filters out any regions of the genome with a much higher read depth than expected to prevent any regions of uninformative/highly repetitive DNA from mistakenly being labeled as insertions.

#### CNV

**Figure 3:**
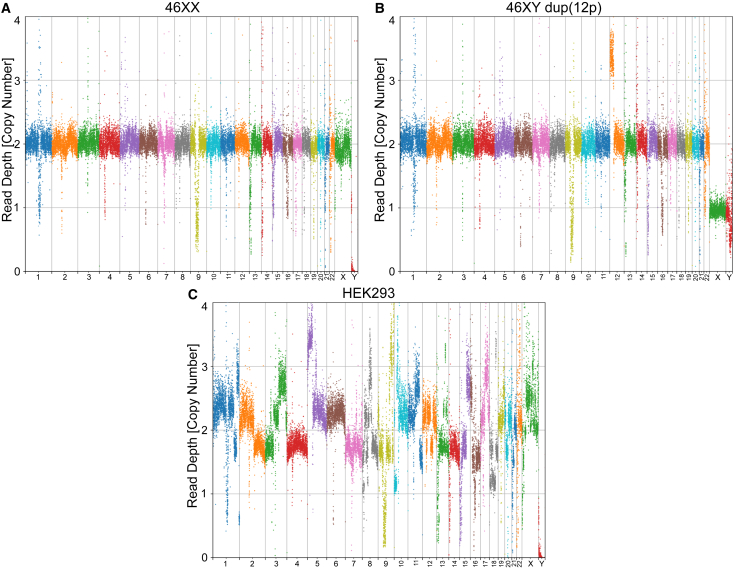
Aneuploidy detection using CNVpytor WGS data from two hiPSC lines (A: female 46XX; B: male 46XY dup(12p)) and HEK293 cells (C) were analyzed using SeqVerify. CNVpytor plots are shown. A shows a normal female karyotype, although some read depth variations are present in repetitive DNA near centromeres and telomeres due to challenges in aligning these sequences. B shows an aneuploid male karyotype. C shows the massive aneuploidy of HEK293 cells.

For CNV detection and analysis, SeqVerify uses the CNVpytor package (Suvakov et al., 2021) and generates a Manhattan plot of the normalized read depth at a default resolution of 100 kbp. These plots are useful for visualizing aneuploidies, as shown in Figure 3. We tested this by comparing WGS data from euploid hiPSCs (Figure 3A) and aneuploid HEK293 cells (Figure 3C). We also serendipitously detected a 12p duplication in one of our hiPSC samples (Figure 3B) that has been previously reported as a common abnormality in human PSCs (hPSCs) (Peterson and Loring, 2014). Besides generating plots, CNVpytor will also call CNVs and output a list of any CNVs found in the sample (see File S3).

Additionally, transgene copy numbers can be estimated using the normalized read depth. Transgene plots are produced using the IGV reports package, which generates an interactive report containing the coverage of the user-selected transgene sequences, as well as the genomic coordinates corresponding to known edits. This is helpful for distinguishing full vs. partial transgene insertions. However, IGV reports may not be compatible with all systems, or there may be issues viewing the interactive HTML report. Therefore, we developed an alternative plotting system using the Python library matplotlib. The internal plotting tool uses the output from running samtools depth on the data to compare the amounts of reads per 30 bp to an estimate of an expected value of reads per 30 bp bin calculated from the average read depth. It then plots these data as a histogram of copy number against bin and makes one of these plots per transgene.

#### Contaminant detection

SeqVerify performs contaminant detection using KRAKEN2 (Wood et al., 2019). After aligning reads to the human reference genome, SeqVerify extracts all read pairs where either the read or its mate is unmapped and runs KRAKEN2 to classify them. We recommend that a KRAKEN database containing human sequences be used (for example, PlusPF-8 which is used as default), since unmapped reads may still contain some human sequences that may otherwise generate false positives. Further analysis to estimate contaminant species abundance is then performed with BRACKEN (Lu et al., 2017).

We validated this contaminant detection by running it on data from 14 hiPSC lines that tested negative for *Mycoplasma* by PCR-based methods and one that tested positive. We found no signs of contamination (less than 50 reads for any bacterial or viral species) in the negative samples, and the positive sample had 23,891 reads mapping to *Mycoplasmopsis arginini* (File S2). Furthermore, we analyzed previously published WGS reads from HEK293 cells (SRA number: SRR18054575) to see if this method could detect adenovirus 5, which was used to transform those cells in their original derivation from fetal tissue. We successfully detected 497 reads mapping to *Adenoviridae*, of which 104 specifically matched human adenovirus 5. Additionally, we found that the HEK293 cells used in that dataset were contaminated with *Mesomycoplasma hyorhinis* (File S2).

#### SNV analysis

SNV analysis requires re-alignment of the reads to an un-edited reference genome. Most human SNV annotation databases (for example, ClinVar) use GRCh38 instead of T2T/CHM13 as their reference genome. Therefore, in the SNV portion of the pipeline, the reads are re-aligned to GRCh38, with BCFTOOLS subsequently used to generate a VCF file of the variants found in the reads (Li, 2011). SeqVerify then annotates the VCF file using SnpEff and SnpSift and (by default) the ClinVar clinical database for further annotation and loss of function and effect prediction (Cingolani et al., 2012a; 2012b). Finally, SeqVerify then takes the annotated VCF file and filters it, generating a human- or machine-readable readout of all mutations above a certain quality score and severity threshold, their effects, genes, any loss of function, and their homozygosity, among other data. An example of such a readout is provided in File S3, which also contains other example output.

#### SNV comparison

After running the main SeqVerify pipeline on at least two samples, the SNV results can be compared using the seqverify --similarity command. This classifies the SNVs according to whether they are shared between samples or specific to one sample. This is implemented using the bcftools isec command, with further processing to compute the concordance between the samples. This comparison is useful to detect potential cell line misidentification or to detect mutations in edited cell lines that were not present in the original cells.

### Optimal read depth for SeqVerify analysis

We benchmarked the SeqVerify pipeline across a range of common read depths (5×, 10×, 20×, 30×, 40×, and 50×) to test its sensitivity. To do this, we sequenced a hiPSC line containing PiggyBac transposon integrations at a genome-wide coverage of 50×. We ran the pipeline’s insertion site and variant calling portions and recorded the insertions and variant calls found at 50× coverage, taking those as our ground truth. We then subsampled the 50× FASTQ files to obtain files with the other desired read depths and ran SeqVerify. Finally, we compared the insertions and variant calls found and computed the percentage of insertions/variant calls in the ground truth data that were present in the subsampled files.

**Figure 4:**
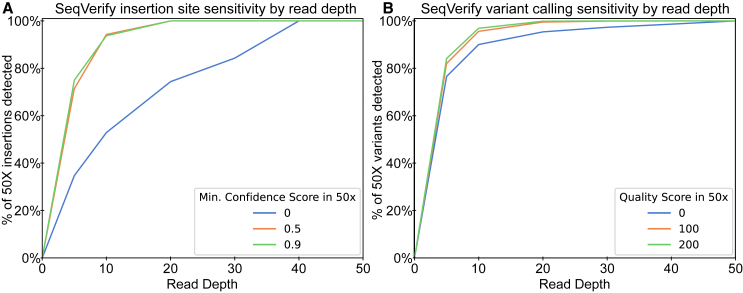
SeqVerify read depth benchmarking (A and B) 50X coverage WGS data from cells containing integrated transposons, and lower-coverage subsamples of that data, were analyzed using SeqVerify. The pipeline was benchmarked based on the minimum read depth at which (A) transposon insertions and (B) variant calls present with high confidence in the 50X data could be recovered.

As can be seen in Figure 4, 20× coverage is sufficient to detect all of the high-confidence (≥0.5 confidence score) insertion sites in the 50× data. Detecting all lower-confidence insertions, which have fewer associated reads, requires 40× coverage. Similarly, the pipeline recovers all of the variant calls in the 50× data at 20× read depth if the variants had a quality score of at least 100 and does not recover the variants until the full 50× data if the variants are not filtered based on quality. Performance with lower-depth WGS data is also very good, with 10× read depth reporting over 90% of high-confidence insertion sites and variant calls, and about 50% of total insertion sites and 90% of total variant calls.

Based on this analysis, we recommend a coverage of at least 20× when using SeqVerify for the detection of variants and untargeted integrations. However, 10× coverage is more economical and allows on-target edit validation as well as the detection of large-scale aneuploidy.

### Benchmarking of SeqVerify on external data

Finally, we evaluated the performance of SeqVerify on a set of 15 wild-type, mutant, and edited hiPSC lines previously analyzed by WGS (at ∼30× coverage), karyotyping, copy-number qPCR, and Sanger sequencing in a separate study (Simkin et al., 2022). In that study, careful manual analysis of WGS data (using a GATK-based approach) and Sanger sequencing was used to determine that four edited lines had unwanted on-target insertions of DNA. SeqVerify was able to automatically report all insertions of plasmid or mitochondrial DNA and also correctly show the outcomes of editing at the target sites (Table 2). No aneuploidy was detected in any of the lines, in accordance with the previous karyotyping results. Therefore, SeqVerify can replicate the results of previous best-practice WGS quality control methods while having the advantage of automatic rather than manual analysis.

**Table 2:** Benchmarking of SeqVerify on independent WGS data from 15 hiPSC lines

Cell line	Average coverage	Previous WGS findings	DNA/plasmid insertions detected?	Correct variants found?
KCNQ2-01-het parental #5	25X	Heterozygous p.T274M; single blocking variant	None	Yes
KCNQ2-01-het parental	25X	Heterozygous p.T274M; single blocking variant	None	Yes
KCNQ2-01-G6	28X	Heterozygous p.T274M; single blocking variant; plasmid DNA insertion; heterozygous 17 bp deletion adjacent to edit site	Yes (plasmid insertion)	Yes
KCNQ2-01-A6	27X	Heterozygous p.T274M; single blocking variant; plasmid and endogenous (human chr4) DNA insertion	Yes (plasmid insertion); insertion of chr4 DNA not automatically reported, but visible in IGV plot	Yes
KCNQ2-04-het parental	28X	Heterozygous p.R581Q	None	Yes
KCNQ2-04-4	27X	p.R581Q corrected to wild type; two blocking variants	None	Yes
KCNQ2-04-55	22X	Heterozygous p.R581Q; mtDNA insertion	Yes (mtDNA insertion)	Yes
KCNQ2-03-het parental	22X	Heterozygous p.R207W	None	Yes
KCNQ2-03-C47	22X	p.R207W corrected to wild type; single blocking variant; mtDNA insertion; heterozygous 10 bp deletion adjacent to edit site	Yes (mtDNA insertion)	Yes
KCNQ2-03-C12	23X	p.R207W corrected to wild type; single blocking variant	None	Yes
DNAJC7-01-WT parental	23X	None	None	Yes
DNAJC7-01-het-18	23X	Heterozygous p.R156X; two blocking variants	None	Yes
DNAJC7-01-het-30	22X	Heterozygous p.R156X; two blocking variants	None	Yes
DNAJC7-01-hom-43	22X	Homozygous p.R156X; twin blocking variants; copy-neutral LOH	None	Yes
DNAJC7-01-WT-9	22X	None	None	Yes

SeqVerify replicated the results of the previous study of editing outcomes (Simkin et al., 2022) and additionally showed that all lines were euploid and free of microbial contamination.

## Discussion

In recent years, WGS has emerged as a powerful tool for genetic research and clinical diagnostics. The continued decline in sequencing prices over time has made WGS an attractive method, and we anticipate this decline will continue in the future. Remarkably, WGS at 10× coverage is now cheaper than karyotyping and cost-competitive with commercial microarray-based services such as KaryoStat. The power of WGS was illustrated by a recent study that analyzed 143 wild-type human embryonic stem cell (hESC) lines for SNVs and CNVs, generating a database of sequence-verified hESC lines (Merkle et al., 2022). However, that study was limited to looking at variation in wild-type cells, relied heavily on manual analysis, and also did not publish alignment and variant calling code. Overall, the lack of convenient data analysis methods has presented a barrier for the routine use of WGS in cell line quality control.

Therefore, we developed SeqVerify, a pipeline to analyze WGS data to validate genetic edits and check for abnormalities, including aneuploidy, mutations, and microbial contamination. SeqVerify is easily installable using Bioconda and provides a start-to-finish pipeline, taking raw sequencing reads as input and outputting quality control data. We have validated the performance of SeqVerify on hiPSC lines edited in-house and showed that our pipeline can replicate results from previously published manual methods of WGS analysis.

### Comparison of WGS with previous quality control methods

#### Validating on-target editing

PCR and Sanger sequencing is cost-effective for initial screening but struggles to detect certain unwanted editing outcomes such as large insertions of plasmid or mitochondrial DNA (Simkin et al., 2022). Additionally, some transgene delivery methods, including lentivirus and PiggyBac transposons, involve the random insertion of transgenes into the host genome. Detecting the insertion sites of these transgenes is important for quality control to ensure that essential host genes are not compromised. Furthermore, if plasmids are introduced into cells for gene editing, unwanted plasmid integration events may occur. Specialized PCR-based methods can efficiently map insertion sites of known DNA sequences (Sherman et al., 2017), although they may miss partial insertions where the primer binding site is not inserted. WGS can identify insertion sites in an unbiased manner; although if multiple transgenes with highly similar sequences are inserted at different sites, short-read WGS cannot always determine which transgene is present at each different insertion site.

#### Detecting deleterious mutations

When establishing a new cell line, it is important to rule out the presence of deleterious mutations. These mutations may be due to off-target editing, or may arise spontaneously. Targeted amplification and sequencing of mutation hotspots or predicted off-target edit sites can provide some information, but may miss important mutations. WGS is the only practical way to detect mutations across the entire genome.

#### Detecting aneuploidy

The traditional method of detecting aneuploidy is by karyotyping, with G-banding or fluorescent *in situ* hybridization. This is effective, but laborious, requiring the preparation and staining of metaphase spreads. Additionally, smaller abnormalities, such as 20q11.21 duplication that is common in PSCs, may be missed with standard karyotyping methods (Markouli et al., 2019; Nguyen et al., 2014).

In recent years, DNA microarrays (for example, Thermo Fisher KaryoStat+) have also been used to detect aneuploidy. These are more sensitive and require only a DNA sample. However, the decrease in cost of WGS over the last few years has made WGS a cost-competitive alternative for this method, with the added benefit of achieving even higher resolution. One drawback of short-read WGS (and especially microarrays) relative to karyotyping is that they are less sensitive at detecting balanced chromosomal translocations. If it is important to detect these translocations (and it may not be, given that they are rare, and typically have mild effects), (Warburton, 1991) then traditional karyotyping or long-read sequencing should be used.

#### Detecting microbial contamination

Testing for microbial contamination, especially *Mycoplasma*, is a critical part of good cell culture practice. This can be done by multiple methods, including PCR and enzyme-based kits. We do not recommend WGS as a routine screening method for contamination, due to higher cost, longer turnaround time, and lower sensitivity than PCR. Nonetheless, WGS data can be analyzed to check for the presence of microbial contamination. In principle, any contaminant with a DNA genome can be detected. This is an advantage over PCR-based methods, which can only detect contaminants matching the PCR primer pairs used.

### Perspective

In conclusion, WGS is an effective “all-in-one” method for detecting the most common abnormalities in cell lines and validating on-target edits. Due to the continual decline of sequencing prices, WGS has become an increasingly cost-effective method (Assou et al., 2020), while also providing much more information than previous quality control methods such as microarray-based karyotyping. We believe that SeqVerify will unlock the potential of WGS for hPSC quality control, and more broadly, for verifying the quality of any cell lines.

### Limitations of the study

The main limitations of SeqVerify are based on its use of short-read DNA sequencing as input. SeqVerify is not set up to detect balanced translocations, and in the case of different transgenes with shared terminal regions, it cannot tell which transgene is present at each insertion site. Long-read sequencing would be a better option in those cases. We also note that the default settings of SeqVerify do not detect variants that are present on alt contigs, and users interested in such variants will need to use a reference genome containing these. Furthermore, epigenetic or transcriptional abnormalities cannot be detected based on only DNA sequence data. Notably, PSCs can have epigenetic abnormalities such as loss of imprinting (Bar et al., 2017) or erosion of X inactivation for female PSC lines (Cloutier et al., 2022). Since these abnormalities do not alter the genomic DNA sequence, they are not currently detectable by short-read WGS technology, so alternative methods should be used to detect them. We are particularly interested in using nanopore sequencing to directly detect DNA methylation for this purpose.

## Experimental procedures

### Experimental model details

#### iPSC culture

Human iPSCs were cultured in mTeSR Plus medium (STEMCELL Technologies) on standard polystyrene culture plates coated with Matrigel (Corning) or Geltrex (Thermo Fisher Scientific). Four lines were used: PGP1 (male) and ATCC-BSX0115, ATCC-BSX0116, and F66 (all female). Cells were passaged as small clumps using 0.5 mM EDTA and treated with 10 mM Y-27632 for 24 h after passage. Cells were routinely tested for *Mycoplasma* using the ATCC Universal Mycoplasma Detection PCR kit. All tested negative, except one sample known to be contaminated with *Mycoplasma* that was used solely as a positive control for the KRAKEN2 analysis.

#### Generation of knockin iPSC lines

Generation of knockin lines was performed as previously described (Pierson Smela et al., 2023). Briefly, homology donor plasmids were constructed by Gibson assembly of a bacterial plasmid backbone, 5′ and 3′ homology arms PCR-amplified from genomic DNA, and a fluorescent protein insert. For all knockins except *SYCP3*, the insert also contained a PGK-PuroTK selection marker flanked by Rox sites, which was excised upon expression of Dre recombinase. Single-guide RNA (sgRNA) oligos targeting the knockin site were cloned into pX330 (Addgene #42230), which expressed the sgRNA and Cas9. Plasmid sequences are given in File S4.

To generate each line, homology donor plasmid and sgRNA/Cas9 plasmid (1 μg each) were co-electroporated into 200,000 hiPSCs using the Lonza 4D nucleofector system with 20 μL of P3 buffer and pulse setting CA-137. The cells were plated in one well of a 6-well plate and, for all knockins except *SYCP3*, selection was begun with puromycin after 48 h. Subsequently, colonies were picked manually with a P20 pipette, transferred to a 96-well plate, and expanded. If required, a further round of electroporation was performed with pCAGGS-Dre to excise the PuroTK selection marker. During this step, selection was performed with ganciclovir (4 μM).

Preliminary genotyping was performed by PCR (primers sequences and gel images are provided in Files S5 and S6). Early-passage lines were cryopreserved using CryoStor CS10 (STEMCELL Technologies). Editing was further confirmed by WGS as described further, which was performed within 20 passages of initial line derivation.

#### DNA extraction and sequencing

Genomic DNA was extracted from hiPSCs using the QIAGEN DNeasy Blood and Tissue kit. 1 million hiPSCs were used per sample. Extracted DNA was submitted to Novogene Corporation for library preparation and Illumina WGS (150 bp paired-end reads to 10× coverage, or 50× coverage for one sample).

#### SeqVerify analysis

SeqVerify analysis was performed for each sample using default settings. Briefly, an augmented reference genome was generated from T2T-CHM13v2.0 containing desired edits as well as transgene sequences for insertion site detection. Reads were aligned to this augmented reference genome using BWA-MEM to generate a BAM file for the validation of on-target editing and detection of transgene insertion sites. The BAM file was then passed as input to CNVpytor for CNV analysis. Unaligned reads were passed as input to KRAKEN2 for microbial contaminant detection. For SNV analysis, reads were aligned to the GRCh38 reference genome and variants were called using bcftools mpileup and bcftools call. Variants were filtered by quality score and annotated using snpEff and snpSift and the ClinVar reference database.

A full description of the SeqVerify pipeline, including instructions on usage, is provided in File S1.

## Resource availability

### Lead contact

Further information and requests for resources should be directed to and will be fulfilled by the lead contact, George M. Church (george_church@hms.harvard.edu).

### Materials availability

This study did not generate new unique reagents.

### Data and code availability

• All code used in this study is available on GitHub at: https://github.com/mpiersonsmela/seqverify
• Raw sequencing reads for hiPSC lines derived from PGP1 are available through the NCBI Sequence Read Archive: PRJNA1019637. In order to respect donor privacy, sequencing data from other hiPSC lines are not publicly available. Sequences of plasmids used for generating knockins are provided in File S3

## Acknowledgments

We thank Dr. Chun-Ting Wu for assistance with validating *Mycoplasma* detection and Dr. Dina Simkin for advice regarding benchmarking. Funding for this project was provided by an NICHD F31 fellowship to M.P.S. (F31HD108898-01A1). Portions of this research were conducted on the O2 High Performance Compute Cluster, supported by the Research Computing Group, at 10.13039/100006691Harvard Medical School.

## Author contributions

Conceptualization, M.P.S.; methodology, M.P.S. and V.P.; software, M.P.S. and V.P.; validation, M.P.S. and V.P.; investigation, M.P.S. and V.P.; resources, M.P.S. and G.M.C.; writing – original draft, M.P.S. and V.P.; writing – review and editing, M.P.S. and V.P.; supervision, G.M.C.; funding acquisition, M.P.S. and G.M.C.; validation, S.L. and E.K.

## Declaration of interests

G.M.C.’s competing interests are listed at: https://arep.med.harvard.edu/gmc/tech.html.

## Supplemental information

File S1. SeqVerify usage guide

File S2. KRAKEN reports

File S3. Example SeqVerify output

File S4. Plasmid sequences

File S5. Oligonucleotides used in this study

File S6. Genotyping gel images

Document S2. Article plus supplemental information

## Footnotes

Supplemental information can be found online at https://doi.org/10.1016/j.stemcr.2024.08.004.
