# Supplementary material for: SeqVerify: An accessible analysis tool for cell line genomic integrity, contamination, and gene editing outcomes
Source: Stem Cell Reports. 2024 Sep 12;19(10):1505–15. doi: 10.1016/j.stemcr.2024.08.004 (PMC11561455; doi:10.1016/j.stemcr.2024.08.004)
Supplement: File S1. SeqVerify usage guide [file mmc1.pdf]

Stem Cell Reports, Volume *19*

Supplemental Information

SeqVerify: An accessible analysis tool for cell line genomic integrity, contamination, and gene editing outcomes

Merrick Pierson Smela, Valerio Pepe, Steven Lubbe, Evangelos Kiskinis, and George M. Church

1. Running SeqVerify

SeqVerify only includes the `seqverify` command, so all calls through the package are done through providing different options to this same base command. The following command is the minimal SeqVerify call that has both untargeted and targeted insertions:

```
seqverify --reads_1 sample_forwards.fastq
--reads_2 sample_backwards.fastq --untargeted transgenes.fa
--targeted commands.txt
```

This requires the forward and reverse reads in FASTQ format, a FASTA file containing the untargeted (transgene) sequences the user wishes to detect the insertion sites of, and a TXT file formatted as a command file to specify what exact insertions were made such that the reference genome can be altered. The command above will not run the KRAKEN2 and variant calling portions of the pipeline, since those options are not enabled by default.

The SeqVerify options, and their default settings, are listed below:

1.2 General Options

- `--output` sets the name of the sample, affecting most output filenames and the folder name. It is set to `output` by default, setting the output folder to be named `seqverify_output`.
- `--reads_1` and `--reads_2` set the paired-read FASTQ (or gzipped FASTQ) source files. Also accepts paths to the files if they're not in the working directory (e.g. for use in research clusters).
- `--genome` takes in the file name of the reference genome to be used for everything except (usually) SNV analysis. If left blank and `--download_defaults` has been run, T2T-CHM13v2.0.
- `--threads` and `--max_mem` regulate performance: the former sets how many threads should be used by the pipeline, the latter puts a cap on memory in the pipeline's most memory-intensive process, Burrows-Wheeler alignment, as well as the Java-based subprocesses that the pipeline uses.
- `--start` allows the user to start and stop at any point in the pipeline; this can be done for core efficiency (if on a job scheduler, run all the single-threaded portions on one job, and all the multi-threaded portions on another), as well as further analysis after an initial portion of the pipeline has been run, and allows for the pipeline to be able to start from the last step completed in the event of technical difficulties. The valid options for `--start` are:
    - o "`all`", the default option, which runs the entire pipeline from the start.
    - o "`beginning`", which runs the pipeline from the start until the alignment step (which it does not execute), creating the relevant folders necessary for SeqVerify to run and creating the transgene-augmented genome and GTF files (useful to generate the GTF file for post-processing analysis after a run of the pipeline that did not generate it).

- “align”, which skips the creation of the augmented genome, starting at the alignment process (useful for cohorts of samples where the user is looking for the same markers/transgenes on the same reference genome).
- “readout”, which skips to the creation of the insertion site readout (useful if the production of the readout or the insertion site processing were skipped in a previous run and the user now desires them).
- “cnv”, which skips to the CNV analysis portion of the pipeline (useful if the user is not interested in the insertion site detection or skipped it on a previous run and wants to come back to it).
- “plots”, which skips to the IGVReports insertion site plot generation (useful if the user wants to refresh their plots without re-running the CNV analysis itself).
- “kraken”, which skips to the KRAKEN contamination detection portion of the pipeline (useful if the user is only interested in contamination or if they skipped it on a previous run).
- “variant”, which only runs the SNV analysis portion of the pipeline (useful as a separate option due to its resource-intensiveness).
- “snp_filtering”, which only runs the SNPEff and SNPSift portions of the pipeline (allows for users to regenerate the annotated VCF files without having to rerun the entire variant-calling portion of the pipeline).

* `--keepgoing` can be set to have the pipeline continue past the initial point set in `--start`, and is off by default (so, by default, if `--start` is not set to ‘all’, the pipeline will execute a single step and then stop).
* `--keep_temp` can be set to keep the temp folder if a user wants to keep the temporary files (including the intermediate SAM files produced during alignment, the coverage map used to compute the CNV analysis, and the FASTQ files for all unaligned reads, among other files.) It is off by default, as these files can take up >100 GB per sample.
* `--download_defaults` downloads the default genomes and databases to the working directory. These are T2T-CHM13v2.0 for use in `--genome`, GHRCh38 for use in SNV analysis, the GTF file for CHM13v2.0, and the 8GB PlusPFP KRAKEN2 database for use with `--kraken`. SeqVerify terminates after downloading these.
* `--config` allows the user to specify a path to a configuration file containing all the SeqVerify parameters. A template for a valid configuration file can be downloaded from the SeqVerify GitHub repository and modified as needed. If `--config` is used, SeqVerify will overwrite any other arguments given to it with the ones present in the configuration file, so a `--config` call should just be `seqverify --config path/to/config/seqverify.config` to avoid any potential issues or conflicts.

1.3 Insertion Site Options

- `--untargeted` sets the names (or paths if not in the working directory) of the FASTA files containing the sequences to detect the insertion sites of (transgenes, plasmids, etc.). Accepts more than one argument, space-separated, if necessary.

- `--targeted` is the name or path to a valid command file for insertion of markers where the insertion site is known. Further details on the construction of a valid command file are given below. Only accepts one command file (but a command file can have multiple commands, so this will not restrict analysis).

- `--gtf` allows for the user to specify a path or name to a valid GTF/GFF3 file for the genome, which will be updated with the exact edits specified in `--targeted`. If left blank and `--download_defaults` has been run, defaults to the GTF (GFF3) file for CHM13v2.0.

- `--granularity` and `--min_matches` set the insertion site detection parameters: the former regulates how wide the window of a single insertion site is (default: 500), and the latter sets how many matches must be present at a single site for the site to appear in the readout (default: 1, but a higher number may reduce false positive alignments due to repetitive DNA or other factors).

- `--mitochondrial`, which, if set, looks for and enables insertion site detection on chrM in the provided genome, for the detection of mitochondrial DNA in the rest of the genome.

- `--stringency`, which sets how stringent the confidence score calculations are: higher values result in lower confidence scores, and lower values result in higher scores (default: 0.005). See Section 4 in this file for more information.

- `--spurious_filtering_threshold`, which sets the threshold probability at which the pipeline considers a number of reads to be a spurious region, and filters it out of the insertion site results. Set by default to 0.00001; no filtering at all will occur if set to 0. See Section 4.1 for more information.

1.4 CNV analysis options

- `--bin_size` can be used to set the bins for the Manhattan plot produced by CNVPytor; anything below the original read length will result in meaningless data (default: 100000).

- `--manual_plots` turns off IGVreports for the coverage plots of the given transgenes, and uses an internal matplotlib-based script instead. Not recommended unless there are issues with installing IGVReports.

1.5 KRAKEN options

- `--kraken` can be set to enable KRAKEN2/BRACKEN analysis, as long as the `--database` option is also enabled and is followed by a path to a valid KRAKEN2 database. If left blank and `--download_defaults` has been run, SeqVerify will use PlusPF-8GB, its default database .

1.6 Variant calling options

- `--variant_calling` can be set to enable SNV analysis on the sample. It takes two additional arguments: the genome to be used to re-align the reads for SNV analysis, as well as the annotation database to use. If left blank and `--download_defaults` has been run, it will re-align the reads to GRCh38 and use the latest version of ClinVar available on its FTP server for annotation.

- `--variant_intensity` sets the minimum severity to be reported in the final readout, out of *MODIFIER* (lowest), *LOW, MODERATE, HIGH* (highest). For example, setting `--variant_intensity` to *MODERATE* will only let variants of *MODERATE* or *HIGH* severity be reported in the final readout. If left blank or not specified, defaults to *MODERATE*.

- `--min_quality` sets a minimum quality filter (using the Phred quality scale) for both variant calling and the similarity detection portion of the pipeline. SNPs above this score will be counted and saved, SNPs below it will be ignored. If left blank, defaults to 100.

- `--variant_window_size` activates only if a command file is specified in `--exact`. If a command file is given, when the pipeline runs through SNV analysis, it will automatically print any variants within `variant_window_size` bases around the start of all commands to the VCF summary file, regardless of their quality or intensity. Set by default to 10000 (thus producing a ±10kb window around every command), turns off the feature completely if set to 0.

- `--similarity` is a three-argument option: it takes in two VCF files and a minimum severity (from the same set as the `--variant_calling`), and returns the Jaccard similarity of the two files for simpler stem cell line identification, filtering for all SNPs above or at the given severity. It can also be paired with the `--min_quality` option to additionally filter based on quality.

2. Command Files

SeqVerify is set up to take exact gene edit sites as inputs, as well as untargeted (usually transgene) integrated sequences, in making the insertion site readout. While untargeted insertions can just be specified by providing the FASTA file of the transgene that the user wants to check for (which will be appended to the genome as an extra nucleotide sequence), to place targeted edits SeqVerify requires some additional information, such as the location of the edit, and whether the edit is a deletion, insertion, or replacement.

This is done through a "command file", a specially formatted text file that the `--targeted` flag takes as its argument. One command is uniquely specified by the name of the chromosome (or other sequence) where the edit is taking place, the start and end coordinates to be deleted (unless the command is a pure insertion), and the sequence to be inserted (unless the edit is a pure deletion, in which case no sequence is required), and every line corresponds to a separate command. Commands are thus of the form "`CHR:START-END SEQUENCE`", where the whitespace between `CHR:START-END` and `SEQUENCE` is a tab character. SeqVerify will delete the bases from START to END exclusive of both (i.e. deleting bases START+1 to END-1). All coordinates will be interpreted as the positive-sense strand. If a sequence is specified, SeqVerify will insert it after deleting the bases from base START+1 onwards. A pure insertion with no deletion can be specified by using the same coordinate for both start and end.

Should there be multiple commands acting on the same chromosome that may influence one another (such as a command deleting 3 bases but inserting 5, which will shift all other commands after the site of the insertion by two bases), SeqVerify will automatically handle change in the base coordinates such that the user does not need to work out the effect that a command will have on other commands themselves.

Command type	Command	Explanation
Deletion	`chr2:0-10`	Delete the first 10 bases in chr2 and not replace them with anything.
Replacement	`chr5:10-20 GCT`	Delete the 11th to 19th bases and replace them with GCT.
Insertion	`chr1:1-1 AGCT`	Not delete anything, and insert AGCT after the first base (i.e. positions 2–5).

Supplementary Table 1: Example Commands

3. Interpreting Output

SeqVerify will output a single folder, `seqverify_output` where *output* is the value of the required `--output` argument, the name of the sample. This will contain at most four subdirectories depending on which portions of the pipeline are performed:

- `insertion`, which contains all files related to the insertion site detection:
    - o `seqverify_output_markers.bam` and its corresponding index, a BAM file containing the reads aligned to the genome with the addition of the transgene sequences.
    - o `seqverify_readout.txt`, the aforementioned readout for insertion site detection. This is a comma-separated file, with headings `chromosome, position, gene, nonchimeric_count, chimeric_count,` and `confidence,` respectively flagging the chromosome the transgene was found on (as a string), its position (as a positive integer), the name of the transgene found (as a string), the number of non-chimeric and chimeric matches (as integers), and the confidence score calculated for that insertion (as a float).
    - o `seqverify_readout.sorted.txt,` the above readout sorted by the chromosome the transgene was found on, with ties being broken by the transgene name in alphabetical order, and then broken again by location if necessary.
    - o `seqverify_output_collated.fa`, the reference genome augmented with all untargeted transgenes as separate chromosomes.

- An IGVReport for graphical viewing of alignments to edit sites and transgene sequences, `igv_viewer.html`.
- If IGVReports was not enabled, a read depth histogram is generated for every chromosome and transgene by matplotlib. These are titled `fig_NAME.png`, where `NAME` is the name assigned to the chromosome/transgene in the FASTA file (e.g. `fig_chr1.png`).

- `copy_number`, containing all of the Copy Number Variation files:
  - `output.pytor`, the CNVPytor binary file, which can be used to generate further plots or further process the CNV data if necessary.
  - `output.global.0000.png`, the Manhattan plot of the copy number across the genome provided.
  - `calls.bin_size.tsv`, all of the CNV calls found by CNVPytor.
- `kraken`, containing the files related to the contamination analysis:
  - `classified_seqs_output.kreport`, a human-readable report of the microbial sequences detected by KRAKEN2.
  - `classified_output.kraken`, the KRAKEN2 binary output files used to generate the report.
  - `classified_seqs_output_1.fq`, `classified_seqs_output_2.fq`, the FASTQ files containing the sequences classified by KRAKEN.
  - `classified_seqs_output.bracken`, the BRACKEN statistical analysis output for the KRAKEN report.
- `variant_calling`, containing the files related to SNP calling:
  - `seqverify_output.ann.vcf`, the database-annotated (ClinVar by default) VCF file output of the SNPs found in the reads provided.
  - `seqverify_output_variants.tsv`, a human-readable file containing information about all mutations above a certain severity and quality threshold.
  - `seqverify_snp_quality.png`, a histogram of the SNP quality scores.

An example of SeqVerify output (excluding BAM and VCF files due to size limitations) is provided as Supplementary File 3.

4. Confidence Score Calculations

The insertion site confidence scores (as described in section 3) were calculated as follows. We assume[23] that the probability that, for some haploid read depth h, the number of times we observe that read in the relevant WGS data follows a Poisson distribution with mean h.

Therefore, to calculate the probability that an insertion site we find in the alignment data is real (which will be our confidence score), we take a Bayesian approach. Define an event R corresponding to the insertion site being real (and notice that its complement R^c implies the insertion site is not real) and some discrete random variable O denoting the number of observed reads. Recalling h as our haploid read depth, note that for some number of observed reads $O=x$, we have $P(O=x|R) \sim Pois(h)$.

Our confidence score in this notation, given the variables above, is equal to $P(R|O=x)$. Thus, by Bayes' Theorem, its formula is:

$$P(O = x) = \frac{P(O = x|R)P(R)}{P(O = x|R)P(R) + P(O = x|R^c)P(R^c)}$$

Note that $P(O=x|R^c)P(R^c)$ is the probability of a false positive: we assume there may be sites in the genome which look similar to the selected transgene sequences, and these register as "matches" in the insertion site detection portion of the pipeline. This error rate depends on the similarity between the transgene sequence and the human genome, and we therefore allow users to adjust it by setting the `--stringency` parameter. The default setting, which works well for most transgenes, is $0.005 \times P(R)$. This simplifies the equation down to the following, which is the default way confidence scores are calculated in SeqVerify:

$$P(O = x) = \frac{P(O = x|R)P(R)}{P(O = x|R)P(R) + 0.005\,P(R)} = \frac{P(O = x|R)}{P(O = x|R) + 0.005}$$

4.1 Spurious Filtering Calculations

The insertion site detection system filters out repetitive sections of DNA as follows. Similarly to the previous section, we assume that the coverage across reads is also Poisson-distributed with mean h (recall h is the haploid read depth of the genome overall).

SeqVerify empirically determines h for the sample provided, and calculates the read depth r^* at which $P(O > x)$ equals the `--spurious_threshold` parameter. Then, if a potential insertion site has more than r^* reads, it is considered a site with a spurious read depth, and filtered out. Reads filtered out in this way do not appear in the final readout.
