# Supplementary material for: SeqVerify: An accessible analysis tool for cell line genomic integrity, contamination, and gene editing outcomes
Source: Stem Cell Reports. 2024 Sep 12;19(10):1505–15. doi: 10.1016/j.stemcr.2024.08.004 (PMC11561455; doi:10.1016/j.stemcr.2024.08.004)
Supplement: File S3. Example SeqVerify output [file mmc3.zip › Supplemental_File_3_Example_output/insertion/igv_viewer.html]

IGV Variant Inspector 


Variants
