# Supplementary figures and images for: SeqVerify: An accessible analysis tool for cell line genomic integrity, contamination, and gene editing outcomes
Source: Stem Cell Reports. 2024 Sep 12;19(10):1505–15. doi: 10.1016/j.stemcr.2024.08.004 (PMC11561455; doi:10.1016/j.stemcr.2024.08.004)

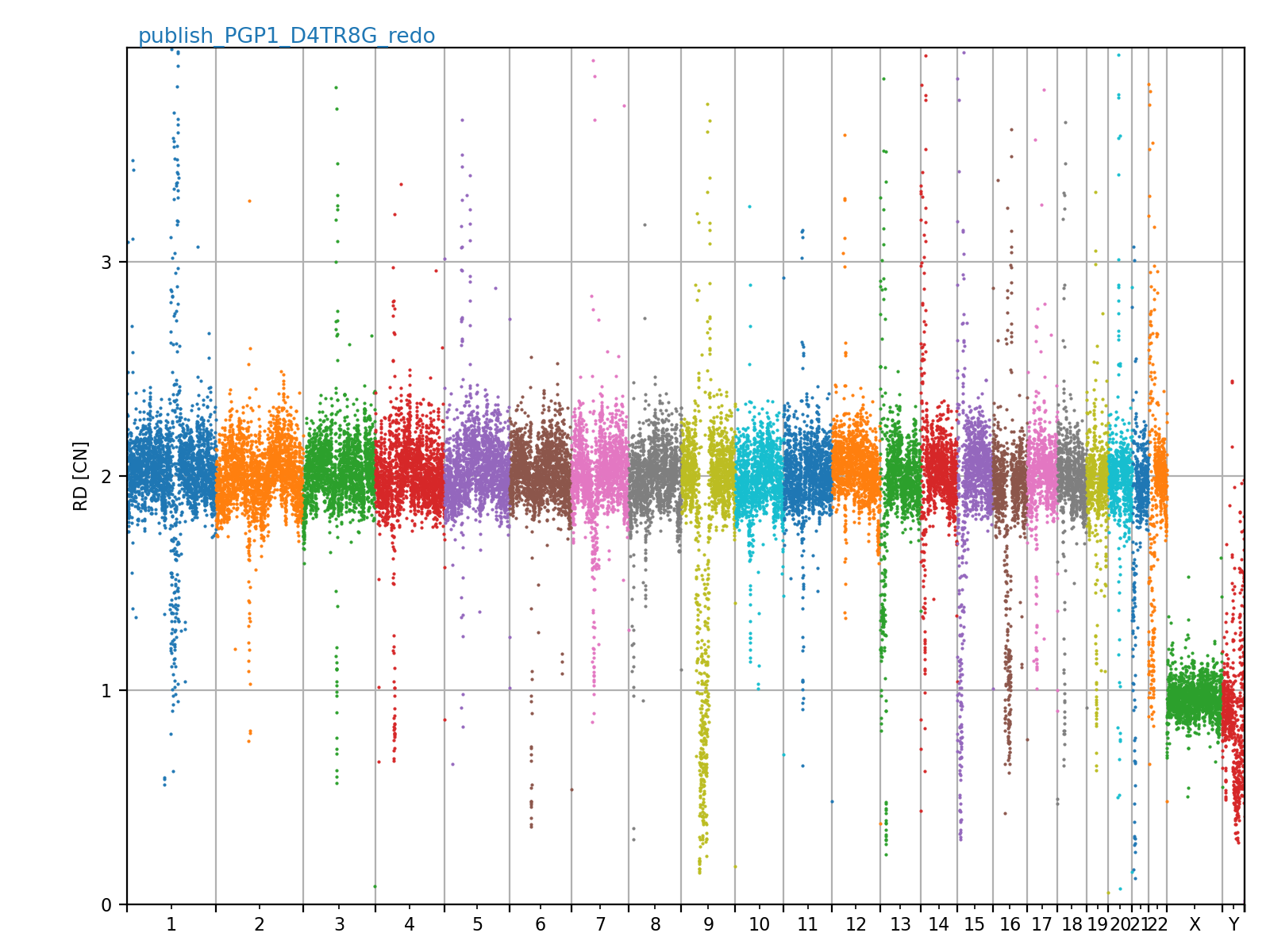

Supplement: File S3. Example SeqVerify output [file mmc3.zip › Supplemental_File_3_Example_output/copy_number/publish_PGP1_D4TR8G_redo.global.0000.png]

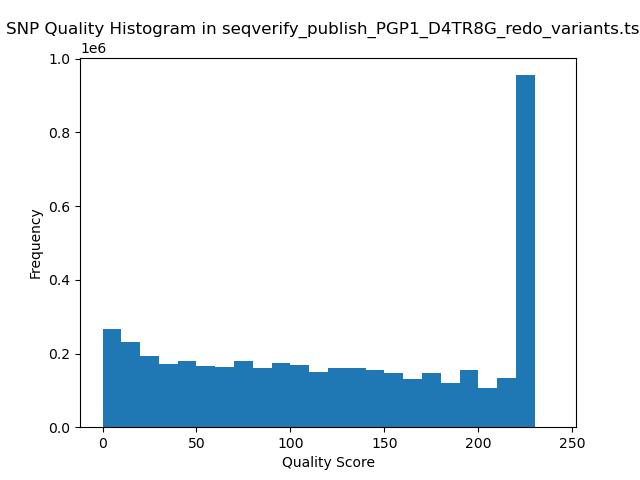

Supplement: File S3. Example SeqVerify output [file mmc3.zip › Supplemental_File_3_Example_output/variant_calling/seqverify_snp_quality.png]

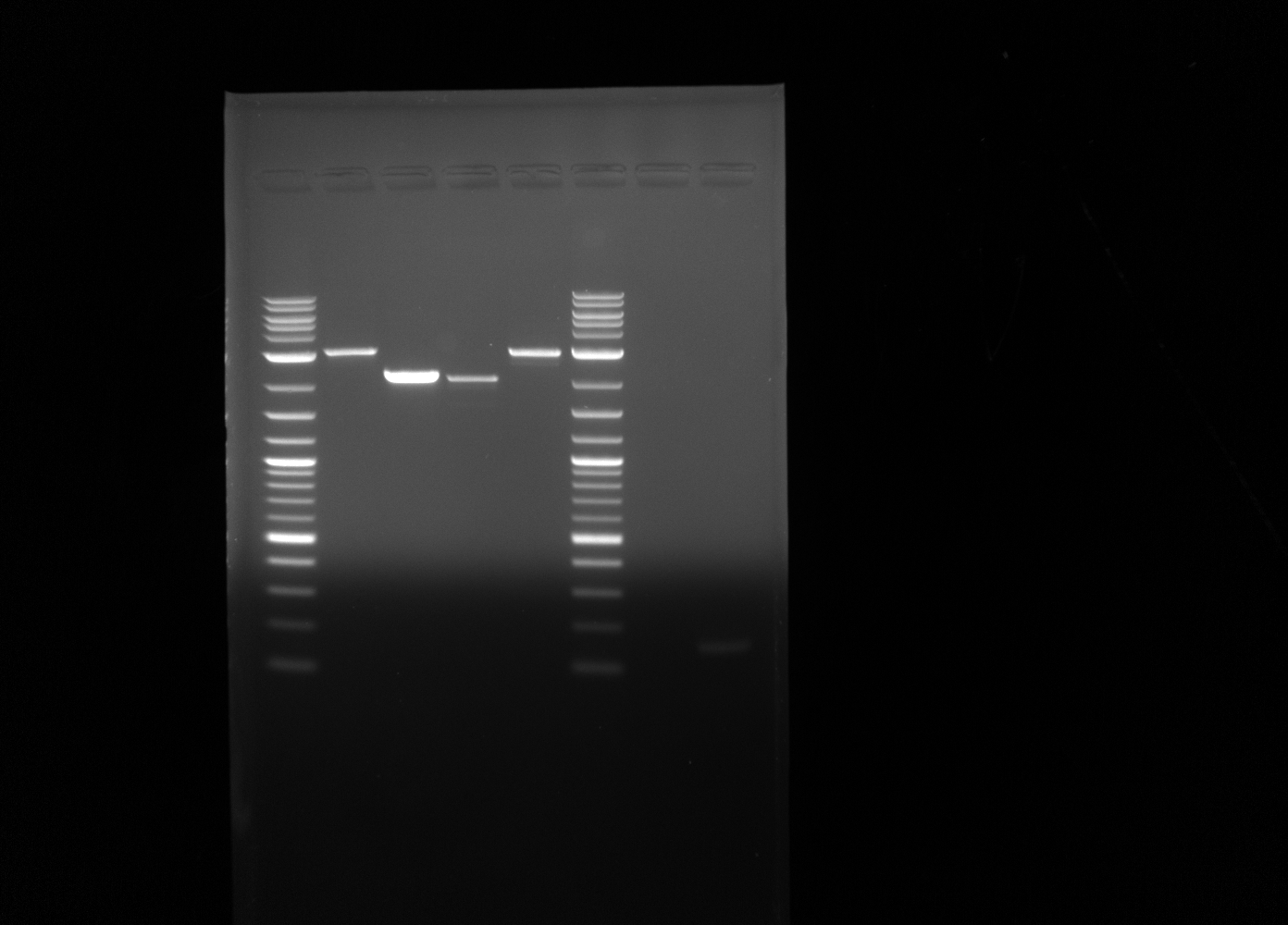

Supplement: File S6. Genotyping gel images [file mmc6.zip › Supplementary_File_6_Gel_Exports/NANOS3 and NPM2 genotyping/2022-12-07_NPM2_NANOS3.png]

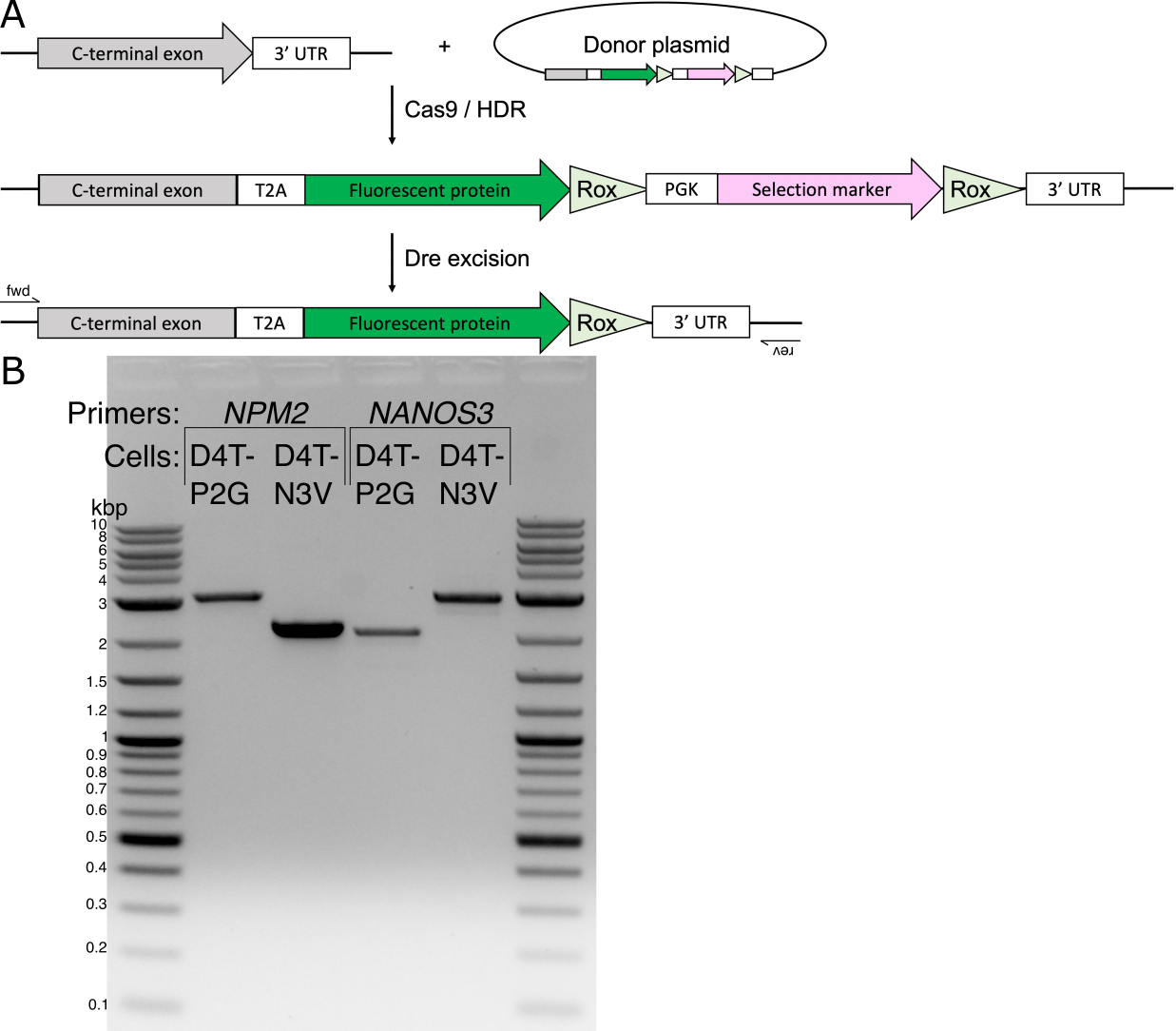

Supplement: File S6. Genotyping gel images [file mmc6.zip › Supplementary_File_6_Gel_Exports/NANOS3 and NPM2 genotyping/2022-12-16_genotyping_fig.png]

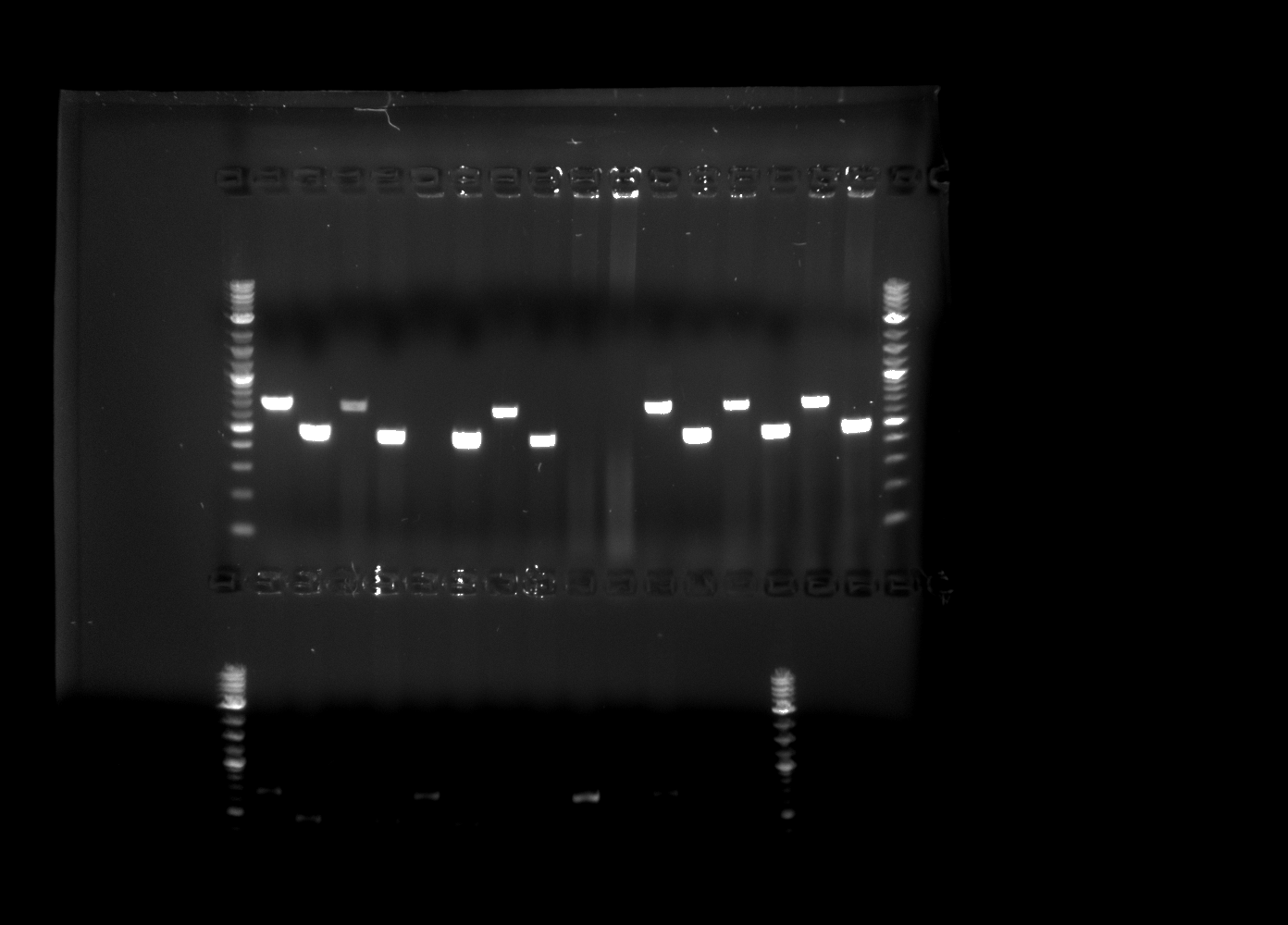

Supplement: File S6. Genotyping gel images [file mmc6.zip › Supplementary_File_6_Gel_Exports/DDX4 genotyping/2022-08-29_PGP1_DDX4_wt_ins.png]

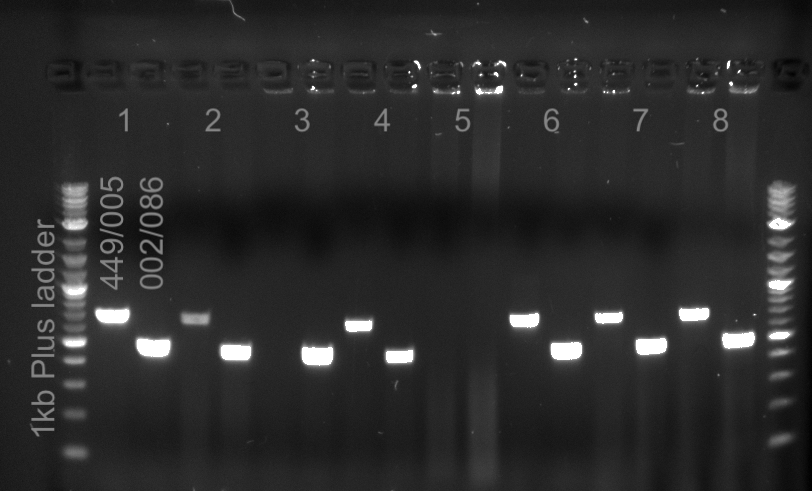

Supplement: File S6. Genotyping gel images [file mmc6.zip › Supplementary_File_6_Gel_Exports/DDX4 genotyping/2022-08-29_PGP1_DDX4_wt_ins_annotated.png]

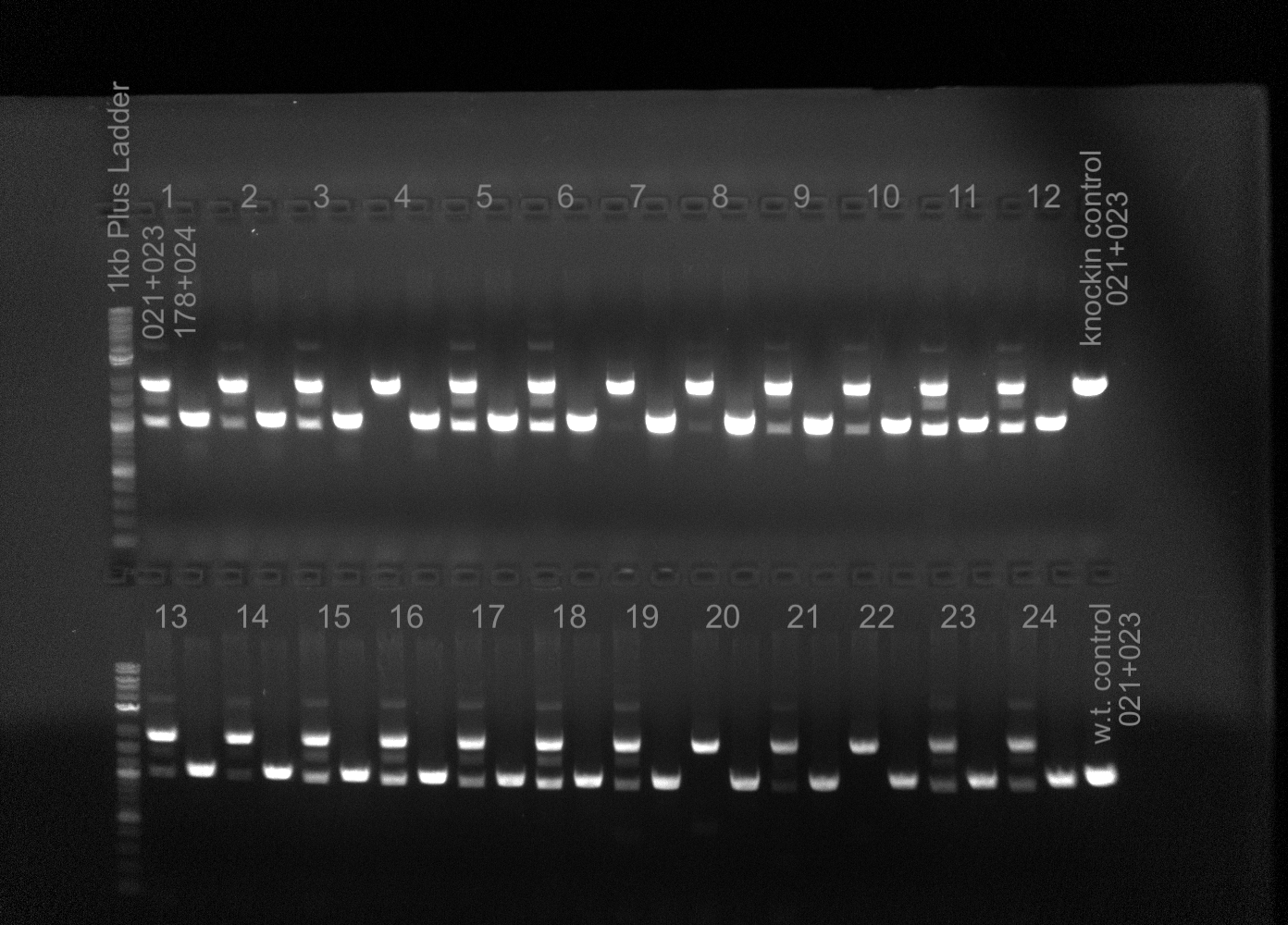

Supplement: File S6. Genotyping gel images [file mmc6.zip › Supplementary_File_6_Gel_Exports/REC8 genotyping/2022-11-07_PGP1_3.2_REC8_Dre_021-023+178-024_annotated.png]

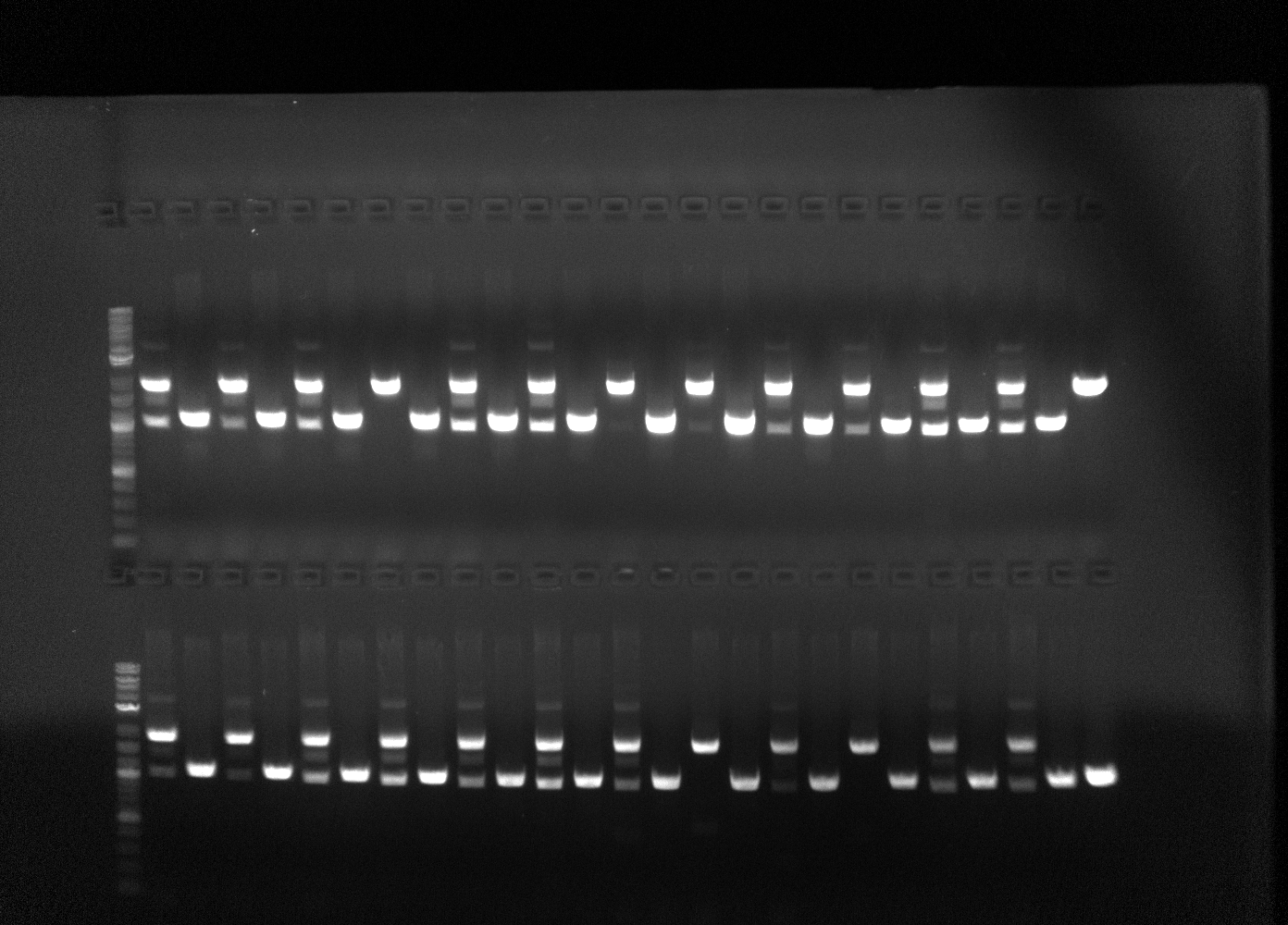

Supplement: File S6. Genotyping gel images [file mmc6.zip › Supplementary_File_6_Gel_Exports/REC8 genotyping/2022-11-07_PGP1_3.2_REC8_Dre_021-023+178-024.png]

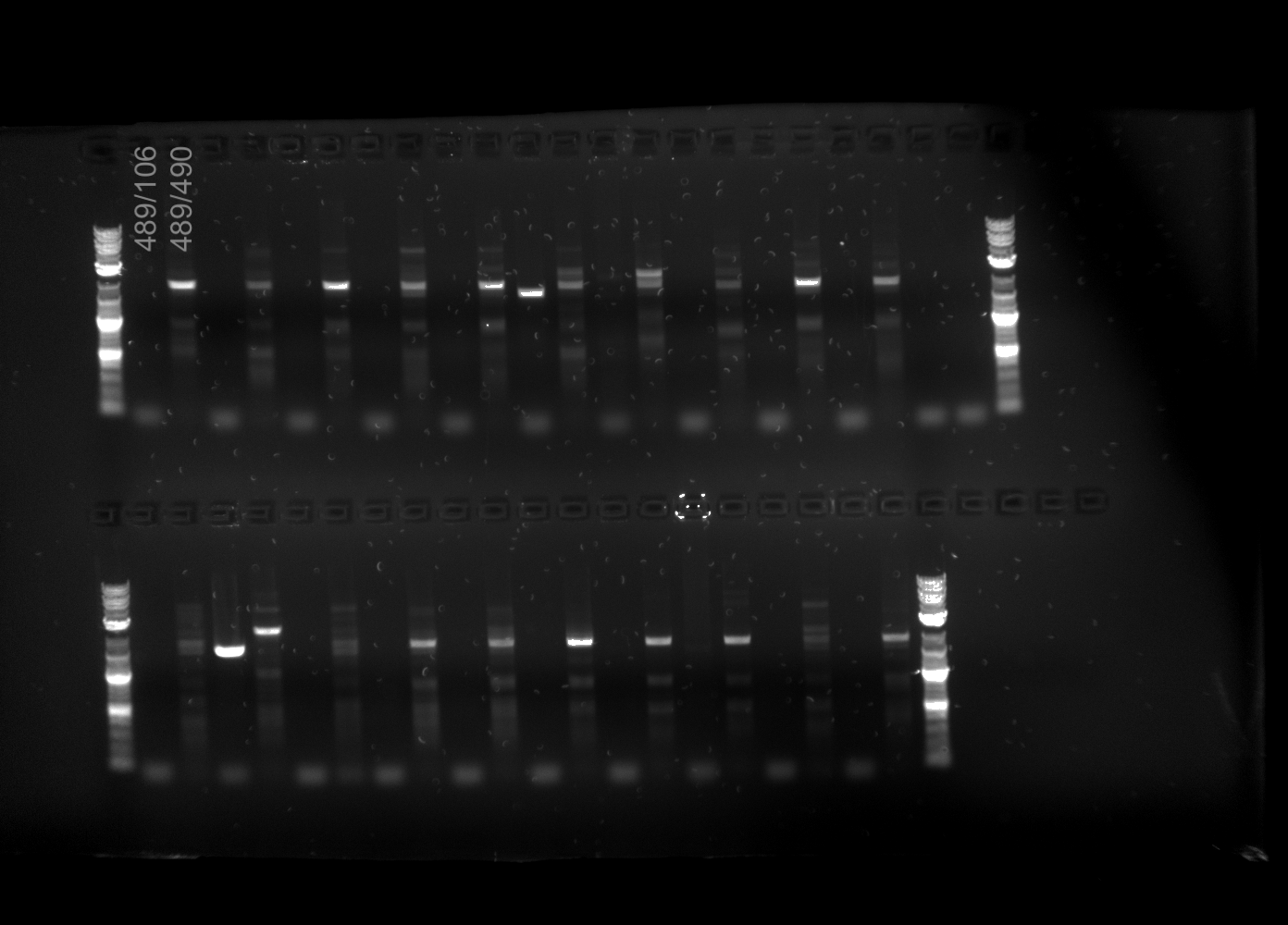

Supplement: File S6. Genotyping gel images [file mmc6.zip › Supplementary_File_6_Gel_Exports/SYCP3 genotyping/2022-12-01_10h30m21s_SYCP3_annotated.png]

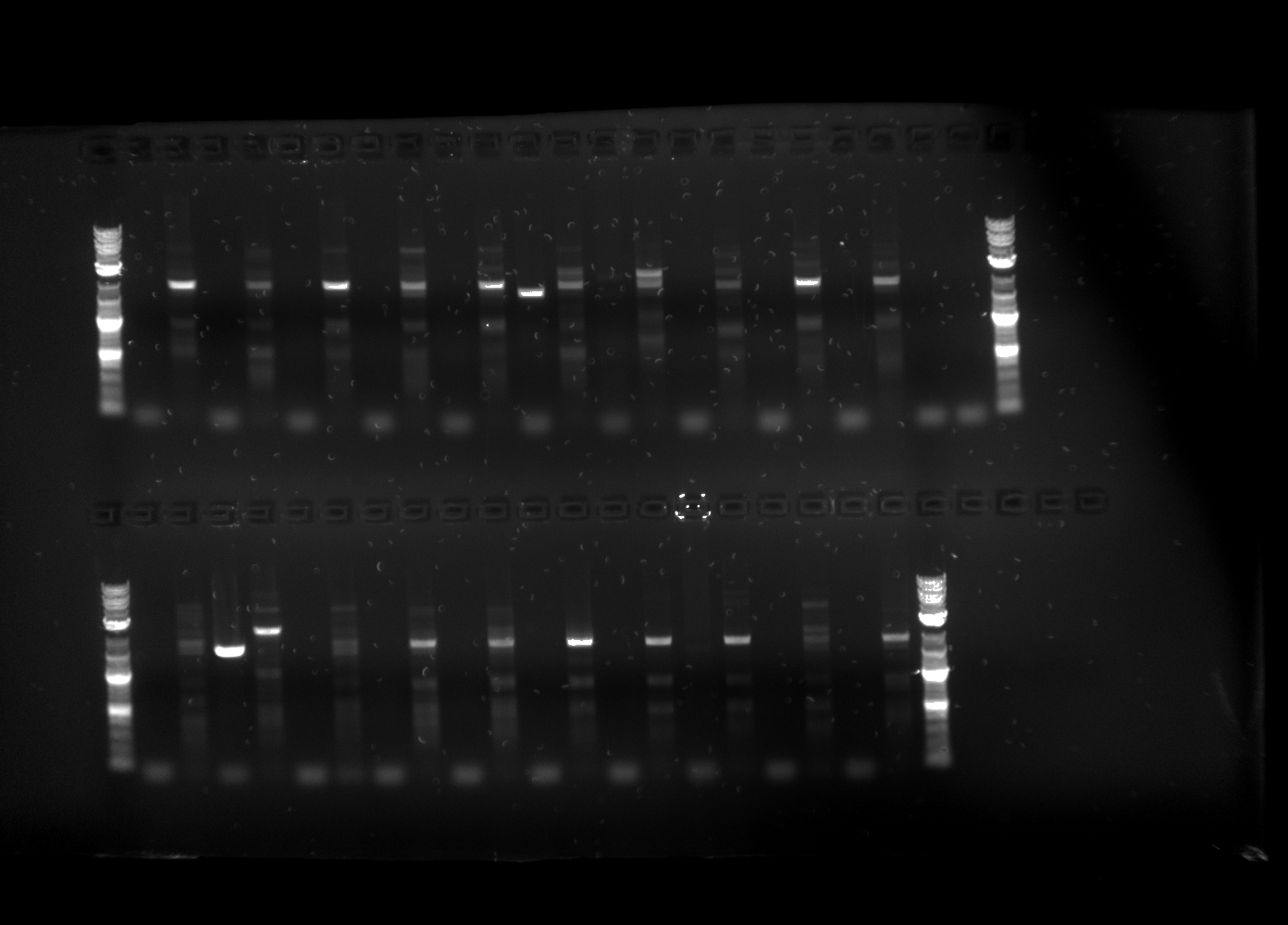

Supplement: File S6. Genotyping gel images [file mmc6.zip › Supplementary_File_6_Gel_Exports/SYCP3 genotyping/2022-12-01_10h30m21s_SYCP3.png]
